# Management of Mandibular Angle Fracture in a 9-year-old with Miniplate and Monocortical Screws: A Clinical Challenge

**DOI:** 10.5005/jp-journals-10005-1471

**Published:** 2017-02-27

**Authors:** Karthik Shunmugavelu, Kumaravel Subramaniam

**Affiliations:** 1 Consultant, Department of Dentistry and Faciomaxillary Surgery, Kasthuri Multispeciality Hospital, Chennai, Tamil Nadu, India; 2 Consultant, Department of Dentistry and Faciomaxillary Surgery, Kasthuri Multispeciality Hospital, Chennai, Tamil Nadu, India

**Keywords:** Mandibular angle, Miniplate, Open reduction and internal fixation, Osteogenesis.

## Abstract

The main characteristic feature of the pediatric mandible is that of decreased dimension, which leads to compromises in the open reduction and internal fixation (ORIF). In the dental segment, the cervically bulbous short stature primary teeth might act an obstacle during the maxillomandibular fixation conventionally. An increased osteogenic potential of bones favors rapid consolidation and remodeling in the affected region. The mixed dentition of the ugly duckling stage adds more burden while stabilizing the fractured segments. The main goal of the clinician is to achieve and restore the facial appearance and function. Hereby, we present a clinical challenge depicting a 9-year-old male with mandibular angle fracture managed by miniplate and monocortical screws fixation.

**How to cite this article:** Shunmugavelu K, Subramaniam K. Management of Mandibular Angle Fracture in a 9-year-old with Miniplate and Monocortical Screws: A Clinical Challenge. Int J Clin Pediatr Dent 2017;10(4):391-393.

## INTRODUCTION

The most common causes of pediatric mandibular fractures are road traffic accidents, fall, sports injury, and violence. The most common site affected in the mandible is condyle, followed by symphysis, parasymphysis, body, and, finally, the angle region.^1^ The gender predilection directs to males rather than females. The age group between 6 and 12 years is commonly involved. The main reason for the incidence corresponds to the reduced cranial dimension at around the age of 6 years and, thereby, progresses gradually.^2^ Fracture management depends on the patient age, site, severity, and comorbidity. The main concept is to restore facial appearance and function, thereby leading to a perfect occlusion.^3^ Factors to be considered during the treatment are avoidance of possible injury to the adjacent teeth, underlying permanent teeth, and growth status because of the mixed dentition stage.^4^ Hereby, we present a clinical scenario of a 9-year-old male with fracture of right mandibular angle fracture, managed with miniplate and monocortical screws for ORIF.

## CASE REPORT

**Fig. 1:**
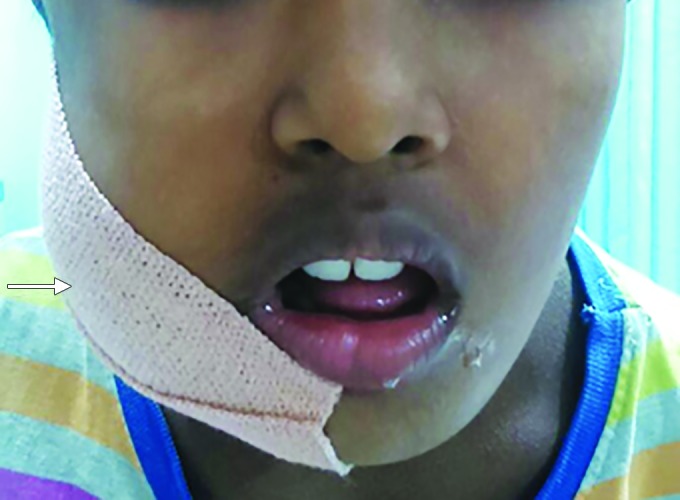
Preoperative clinical view (arrowhead) with swelling in relation to right angle of mandible

A 9-year-old male presented with a swelling on his right side of the face that had been present for 2 days, in the Department of Dentistry and Maxillofacial Surgery. He also said that he experienced difficulty and pain during mouth opening. The case history revealed that the patient had a fall from the vehicle in which he traveled. On extraoral examination, swelling was seen extensively for right preauricular region to right inferior border of the mandible. Swelling was of fluctuant nature, warmth present, tender on palpation, and step deformity felt near soft tissue, a part of right mandibular angle region, and mandibular deviation observed. Intraoral examination revealed that his occlusion was deranged and also reduced mouth opening, in the mixed dentition phase. The patient was conscious, oriented, afebrile, coopera-five, and with absence of vomiting or seizures (Fig. 1).

**Fig. 2:**
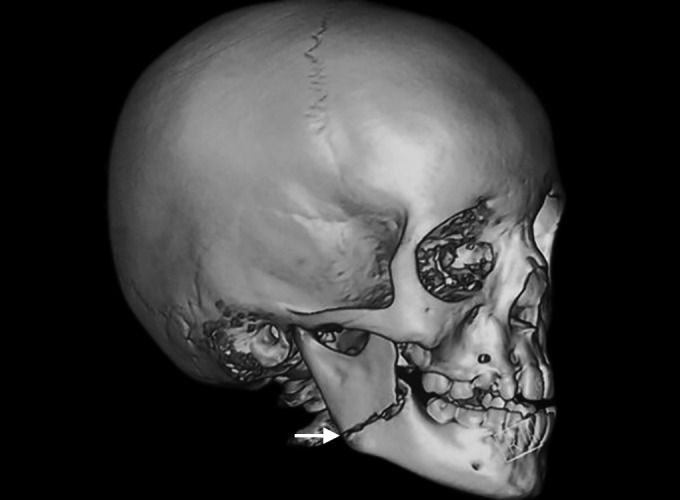
Three dimensional facial CT (arrowhead) with horizontally favorable fracture in relation to right angle of mandible

**Fig. 3:**
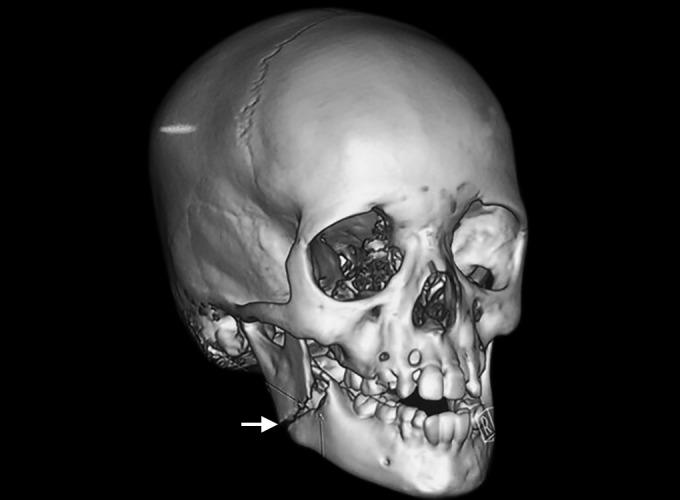
Three dimensional facial CT (arrowhead) with fractured segments

**Fig. 4:**
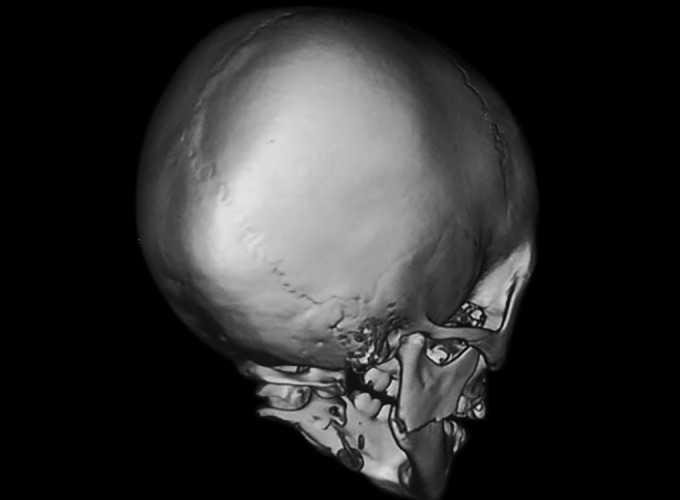
Three dimensional facial CT (arrowhead) depicting displaced right angle of mandible

**Fig. 5:**
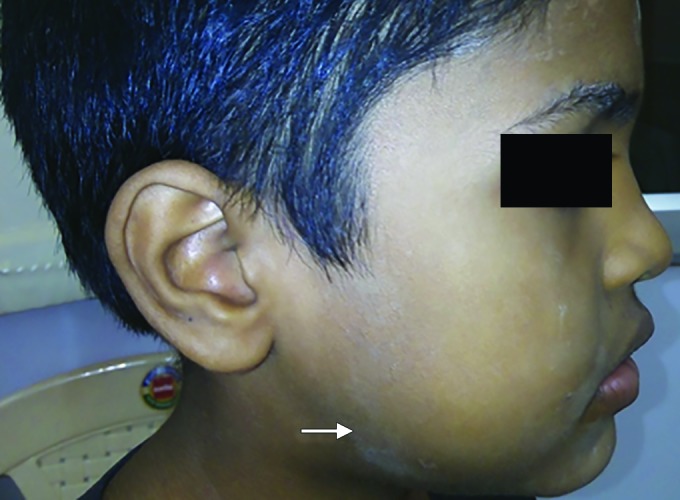
Postoperative (arrowhead) clinical view

Three-dimensional facial computerized tomography (3D facial CT) revealed horizontally favorable fracture of right mandibular angle region (Figs 2 to 4). Clinical findings, 3D facial CT, and treatment plan were explained to the parents and informed consent was obtained. Followed by nil per oral protocol, under general anesthesia, nasal intubation was planned, preparation with 5% povidone iodine, and sterile draping was done. Intraoral approach was done via retromolar incision in the right side. The fracture site was exposed in relation to right angle of mandible, reduced, and kept in occlusion. The fractured site was fixed with titanium miniplates of 2 mm, 4 hole straight plate with gap, and 2 × 8 mm four monocorti-cal screws were used. Flap closure was approximated with 4-0 Vicryl. Hemostasis was achieved. Postoperative recovery was uneventful (Fig. 5). Intraoral wound healing was good followed by stable occlusion and improved mouth opening.

## DISCUSSION

A protective social environment and supervision by parents play an important role in the pediatric facial bone injuries. The most common etiologies for facial trauma in the pediatric category are road traffic accidents, fall, sports injury, and interpersonal altercation. Condyle is the most commonly affected followed by symphysis, body, and angle of the mandible.^2^ Due to decreased size of the cranium around the age of 6 years, mandible is more prone to injury. The predilection targets males in the age group of 6 to 12 years.^2^ Mixed dentition stage, due to its instability, is witnessed in the age group. Modification of the miniplate protocol was developed by Champy and Lodde,^5^ which was earlier presented by Michelet in 1973. Stable fixation is required in this stage in order to avoid further injury to the developing dentition and growth. Discrepancies in the occlusion and alignment are corrected by rapid remodeling and healing properties. Displaced mandibular fractures witnessed in the pediatric category are managed by ORIF.^6^ The fractured segments of the bone undergo gradual consolidation and remodeling due to slow, gradual, and increased masticatory forces. The main advantages of ORIF are reduced treatment span, 3D reconstruction, and primary bone healing.^7^ The fracture repair is controlled by age of the patient, site of fracture, severity of the condition, and the approach used.^8-10^ The major advantage of intraoral approach is the absence of visible scar formation.^11,12^ The ORIF plays an important role in restoration of the lost dental hygiene and dietary habits. Absence of intermaxillary fixation despite ORIF aids in reduced immobilization time, decreased muscular atrophy in conjunction with improved oral hygiene measures, thereby leading to favorable healing period.^13-15^ The handling nature of the metallic plates helps in the ORIF of displaced fractures.^16-18^ Follow-up was done along with counselling of parents regarding futuristic growth-related disturbances, if any.

## References

[1] Srinivasan I, Kumar N, Jaganathan U, Bhandari A (2013). Miniplate for osteosynthesis in a 9-year old with symphysis fracture: clinical report.. Int J Clin Pediatr Dent.

[2] Posnick JC, Wells M, Pron G (1993). Paediatric facial fractures: evolving patterns of treatment.. J Oral Maxillofac Surg.

[3] Hogg NJ, Horswell BB (2006). Clinical practice.. J Can Dent Assoc.

[4] Kushner GM, Tiwana PS (2009). Fractures of the growing. mandible. Atlas Oral Maxillofac Surg Clin North Am.

[5] Champy M, Lodde JP (1976). Localisation des syntheses en function des contraintes mandibulaires.. Rev Stomatol.

[6] Zimmermann CE, Troulis MJ, Kaban LB (2006). Padiatric facial fractures; recent advances in prevention, diagnosis and management.. Int J Oral Maxillofac Surg.

[7] Feller KU, Richter G, Schneider M, Ecklet U (2002). Combination of microplate and miniplate for osteosymthesis of mandibular fractures: an experimental study.. Int J Oral Maxillofac Surg.

[8] Eppley BL (2005). Use of resorbabale plates and screws in pediatric facial fractures.. J Oral Maxillofac Surg.

[9] Haug RH, Foss J (2000). Maxillofacial injuries in the pediatric patient.. Oral Surg Oral Med Oral Pathol Oral Radiol Endod.

[10] Gassner R, Tuli T, Hachl O, Moreira R, Ulmer H (2004). Craniomaxil-lofacial trauma in children: a review of 3,385 cases with 6,060 injuries in 10 years.. J Oral Maxillofac Surg.

[11] Heitsch M, Mohr C, Schettler D (1990). Indications for the surgical treatment of midfacial fractures in children.. Dtsch Zahnarztl Z.

[12] Smartt JM Jr, Low DW, Bartlett SP (2005). Pediatric mandible.. Plast Reconstr Surg.

[13] Yarington CT Jr (1977). Maxillofacial trauma in children.. Otolaryngol Clin North Am.

[14] Gawelin PJ, Thor AL (2005). Conservative treatment of pediatric mandibular fracture by the use of orthodontic appliance and rubber elastics: report of a case.. Dent Traumatol.

[15] Chen CM, Chen YR (1990). mandibular fractures in children-immediate reduction and fixation with orthodontic resin.. Changgeng Yi Xue Za Zhi.

[16] Anderson PJ (1995). Fractures in the facial skeleton in children.. Injury.

[17] Hardt N, Gottsauner A (1993). The treatment of mandibular fractures in children.. J Craniomaxillofac Surg.

[18] Siegel MB, Wetmore RF, Potsic WP, Handler SD, Tom LW (1991). Mandibular fractures in the pediatric patient.. Arch Otolary-ngol Head Neck Surg.

